# The impact of changes in intensive care organization on patient outcome and cost-effectiveness—a narrative review

**DOI:** 10.1186/s40560-016-0207-7

**Published:** 2017-01-25

**Authors:** Alexander F. van der Sluijs, Eline R. van Slobbe-Bijlsma, Stephen E. Chick, Margreeth B. Vroom, Dave A. Dongelmans, Alexander P. J. Vlaar

**Affiliations:** 1 Department of Intensive Care Medicine, Academic Medical Center, Room C3-343, Meibergdreef 9, Amsterdam, 1105 AZ The Netherlands; 2 Department of Intensive Care Medicine, Ter Gooi Ziekenhuizen, Hilversum, The Netherlands; 3 INSEAD Healthcare Management Initiative, INSEAD, Fontainebleau, France

**Keywords:** Intensive care department, Critically ill, Lean, Management, Organization, Total quality management, Six Sigma, Pooling, Closed and open format

## Abstract

The mortality rate of critically ill patients is high and the cost of the intensive (ICU) department is among the highest within the health-care industry. The cost will continue to increase because of the aging population in the western world. In the present review, we will discuss the impact of changes in ICU department organization on patient outcome and cost-effectiveness. The general perception that drug and treatment discoveries are the main drivers behind improved patient outcome within the health-care industry is in general not true. This is especially the case for the ICU department, in which the past decades’ organizational changes were the main drivers behind the reduction of ICU mortality. These interventions were at the same time able to reduce cost, something which is rare for drug and treatment discoveries. The organization of the intensive care department has been changed over the past decades, resulting in better patient outcome and reduction of cost. Major changes are the implementation of the “closed format” and electronic patient record. Furthermore, we will present possible future options to improve the organization of the ICU department to further reduce mortality and cost such as pooling of dedicated ICU into mixed ICU and embedding business strategies such as lean and total quality management. Challenges are ahead as the ICU is taking up the largest share of national health-care expenditure, and with the aging of the population, this will continue to increase. Besides future improvements of organizational structures within the ICU, the focus should also be on the implementation of and compliance with proven beneficial organizational structures.

## Background

Intensive care units (ICUs) are among the most complex and expensive departments in a hospital. The innate complexity of the ICU makes organizational structuring of care an attractive target for performance improvement strategies. One improvement in the past is assigning “intensivists” (specialists in critical care medicine) in managing ICU patients instead of specialists from the referral medical departments, which is also called “closed format” ICU departments. This closed format transformation has shown a beneficial impact on patient outcomes in a number of studies [1–3]. However, due to an aging population and the increasing acuity of illness of hospitalized patients, both the total number of ICU patients and their proportional share of hospital admissions overall are expected to keep growing [4]. Furthermore, although mortality rates have significantly reduced after assigning intensivists to these patients, mortality rates are still relatively high, at up to 28% for a general ICU population [5]. This implies that additional improvements in survival of patients and cost-effectiveness of the ICU departments are necessary. In the present review, an overview will be given on changes in organizational models in the ICU departments in the past and their impact on quality of patient care and cost. Furthermore, possible future improvements will be discussed.

### Methods—systematic search of the literature

The Medline database was used to identify Medical Subject Headings (MeSH) to select search terms. In addition to MeSH terms, we also used free-text words. Search terms referred to aspects of the ICU organization (“Intensive Care department”, “organization”, “management”, “staffing”) as well as related topics (“cost reduction”, “patient outcome”, “lean”, “Toyota”, “total quality management”, “Toyota Production System”, and “monitoring”). Relevance of each paper was assessed using the online abstracts. In addition, the reference lists of retrieved papers were screened for potentially important papers.

### The cost of intensive care health-care services

The intensive care department is the most expensive hospital department. In the US alone, annual critical care medicine costs nearly doubled from 2000 to 2010 (from $56.6 to an estimated amount of $108 billion). Although the proportion of hospital cost allocated to critical care medicine (13.2%) decreased by 1.5% and the proportion of national health expenditures (4.14%) remained stable, the proportion of the gross domestic product used by critical care medicine increased from 0.66% in 2005 to 0.74% in 2010 [6, 7]. In highly developed European health-care systems, the average cost per ICU patient is around €1200 per day and €17,000 per admission [8, 9]. The main drivers of cost are personnel cost followed by infrastructure and pharmaceutical expenditure. This illustrates that reduction of cost should be aimed at the improvement of the utilization of personnel, processes, and infrastructure. We will now discuss the interventions in the past on organizational structures in the ICU department and the impact on patient outcome and cost.

### The impact of the intensivist

#### Closed format vs. open format

In the early days of the ICU, patients admitted to the ICU were treated by the referring physicians. Although referring physicians had extensive knowledge on the specific disease the patient was suffering from, there was a lack of knowledge concerning the intensive care treatment these patients needed. Another issue was the lack of continuity of care for these critically ill patients, as the referring physicians were not providing 7-day-per-week coverage of care. This issue was recognized in the 1990s and resulted in the development of the “Leapfrog initiative” [10]. In short, the Leapfrog initiative proposed that the “intensivist” (specialists in critical care medicine) is present in the ICU during the daytime hours 7 days/week, with no other clinical duties during this time. Hence, the intensivist was assigned to manage ICU patients rather than specialists from the referral medical departments. This closed format transformation has shown a beneficial impact on patient outcomes in a number of studies (see Table 1). A systematic review on the effect of physician staffing on the ICU showed that the assignment of intensivist to the ICU led to an overall 0.61 relative risk (RR) (95%CI 0.50–0.75) reduction of ICU mortality [11]. Furthermore, the assignment of intensivists in some studies led to a reduction of intensive care stay and hospital stay, and, subsequently, a reduction of costs. Of interest, the reduction of cost was only reported in 3 out of 15 studies and suggested a 36 to 61% reduction. Overall, it can be suggested that a closed format staffing is preferable above a traditional ICU staffing. In line with this, the Critical Care Societies recommend the closed format above the “open format.” Although it is recommended, the closed format is still not implemented widely which may be caused by a shortage of intensivists [12, 13].

**Table 1:** The impact of the intensivist on patient outcome and cost in the adult intensive care unit

Reference	Country	Design	Population	Year	Reduction in ICU LOS	Reduction in hospital LOS	Reduction in hospital mortality	Reduction in cost
Multz [1]	USA	Prospective	Medical	1998	Yes, <0.0001	Yes, <0.01	No	n/a
Dimick [2]	USA	Retrospective	Surgical	1994–1998	n/a	Yes, <0.05^a^	Yes, <0.001	Yes, 61%
Carson [3]	USA	Prospective	Medical	1996	No	No	No	n/a
Li [4]	USA	Retrospective	Mixed	1984	No	n/a	Yes, 0.01^a^	n/a
Reynolds [5]	USA	Retrospective	Medical	1986–1988	No	No	Yes, <0.01^a^	n/a
Brown [6]	Canada	Retrospective	Mixed	1984–1986	n/a	n/a	Yes, <0.01^a^	n/a
Manthous [7]	USA	Retrospective	Medical	1992–1994	Yes, <0.05	Yes, <0.05	Yes, 0.002^a^	n/a
Pronovost [8]	USA	Retrospective	Surgical	1994–1996	Yes, <0.05^a^	Yes, <0.05^a^	Yes, 0.05^a^	n/a
Baldock [9]	UK	Prospective	Mixed	1995–1998	n/a	n/a	Yes, 0.001	n/a
Rosenfeld [10]	USA	Prospective	Surgical	1996–1997	Yes, <0.01	No	Yes, 0.008^a^	Yes, 36%
Diringer [11]	USA	Retrospective	Neuro	1996–1999	Yes, <0.05	Yes, <0.05	Yes, 0.001	n/a
Blunt [12]	UK	Retrospective	Medical	2000	No	No	Yes, 0.001^a^	n/a
Hanson [13]	USA	Retrospective	Surgical	1994–1995	Yes, <0.05	Yes, <0.05	No	Yes, not quantified
Ghorra [14]	USA	Retrospective	Surgical	1996	No	n/a	n/a	n/a
Tai [15]	Singapore	Prospective	Medical	1993–1994	Yes, 0.01	No	n/a	n/a

*ICU* intensive care unit, *LOS* length of stay, *USA* United States of America, *n/a* not applicable

^a^Remains significant after adjustment for baseline disease severity

#### Twenty-four-hour availability of the intensivist

The positive effect of the closed format intensive care staffing on patient outcome, length of stay, and cost raised the question whether a 24-h availability of an intensivist would even further improve these results compared to having only an in-house intensivist during office hours. This hypothesis was supported by a study on the neonatal ICU showing mortality reduction among premature neonates admitted when they introduced a neonatologist or neonatal fellow in-house [14]. Another supportive finding was that patients with an acute condition admitted during weekends have worse outcomes than patients with the same diagnoses admitted during the week [15]. Other studies showed that having a 24-h mandatory staffing presence in the ICU further improves processes of care and staff satisfaction and decreases ICU complication rate and hospital length of stay (LOS) [16]. A recent study showed, however, that the beneficial impact of 24-h staffing on mortality reduction is only present in an open format ICU and absent in a closed format staffing ICU [17]. This notion is supported in a randomized trial conducted in an academic medical ICU in the USA, showing no significant effect of nighttime staffing on the length of stay or on ICU mortality compared to day and evening time intensivist staffing [18]. One may question whether it is cost-effective for a closed format ICU as mortality reduction was not present and 24-h staffing will increase personnel expenditure.

#### Intensivist-to-bed ratio

Intensivist specialists are scarce recourses and should be utilized as high as possible. For this reason, Dara et al. looked at the optimal intensivist-to-bed ratio [19]. Four time periods based on intensivist-to-ICU-bed ratios of 1:7.5, 1:9.5, 1:12, and 1:15 were identified. Patients in all time periods did not differ on disease severity. Differences in intensivist-to-ICU-bed ratios, ranging from 1:7.5 to 1:15, were not associated with differences in ICU or hospital mortality. However, a ratio of 1:15 was associated with increased ICU LOS.

### The impact of the ICU nurse

What is true for the intensivist-to-bed ratio is probably also true for nurse-to-bed ratio. In many countries, intensive care nurses are specialized nurses with extra training. It is not known what the optimum ratio is. In most counties, the ratio of nurse to patient is between 1:1 and 1:2. For patients after hepatectomy or esophagectomy, there is evidence that less patients per nurse results in a decrease in pulmonary or infectious complications, while mortality was not significantly different [20]. A recent report from the UK showed that higher nurse workload was associated with higher mortality in a general ICU population [21]. We suggest prospective studies are needed to assess the true optimal nurse-to-bed ratio.

### The impact of outreach teams

Given the fact that critically ill patients are often admitted to the ICU from general nursing departments, it seemed logical to install teams that reach out to these patients before detoriation begins [22]. The Cochrane Collaborative systemically reviewed the literature on the impact of critical care outreach on patient outcomes [23]. Nearly 5000 studies were identified as being potentially relevant with only two randomized controlled trials meeting the inclusion criteria. The first study was the Medical Early Response Intervention and Treatment (MERIT) study, performed in Australia. This was a randomized cluster-controlled trial to study the effects of the introduction of an outreach team. They found that the introduction did not significantly reduce the incidence of unexpected deaths, cardiac arrests, and unplanned ICU admissions [24]. Priestly et al. introduced a nurse-led outreach service that ran 24 h a day and focused on education, support, and practical help for ward staff. This randomized trial resulted in reduced hospital mortality with a trend towards an increased length of stay [25]. The overall conclusion of the Cochrane Review, however, was that the evidence for effectiveness of these outreach services was inconclusive. In a recent multicenter trial, introduction of nationwide implementation in the Netherlands of rapid response systems was associated with a decrease in the composite end point of cardiopulmonary arrests, unplanned ICU admissions, and mortality in patients in general hospital wards [26]. These findings support the implementation of rapid response systems in hospitals to reduce severe adverse events.

### The impact of patient digital management systems

The introduction of patient digital management systems (PDMS) provided intensivists with a fast overview of patient’s critical data. Having an interface with the electronic medical record at the unit level was significantly associated with a lower risk of mortality in the ICU [27]. The finding that electronic medical records integrated with ICU information systems are associated with lower in-hospital mortality adds support to existing evidence on organizational characteristics associated with in-hospital mortality among ICU patients. The PDMS may also have a role in risk predictions. Risk prediction can be implemented for adverse effects of treatments performed on the ICU, e.g., onset of ventilator-induced lung injury while patients are on the mechanical ventilation. An algorithm can easily been built and screen all electronic records and subsequently warn the attending intensivist in case of increased risk [28–31]. The PDMS can also be used to predict risks after ICU discharge. A recent study used the PDMS to validate an automatic risk of unplanned readmission (Stability and Workload Index for Transfer (SWIFT)) calculator in a prospective cohort of consecutive ICU patients [32]. The authors showed and concluded that the PDMS accurately calculates SWIFT score and can facilitate ICU discharge decisions without the need for manual data collection. Another area where the PDMS resulted in increased patient safety is medication prescription. Electronic prescribing results in increased medication safety [33]. In conclusion, the introduction of the PDMS has resulted in a better patient outcome and reduction of the intensivist task load by which the utilization of intensivists becomes optimized.

### Possible future improvements

#### Pooling of ICU departments

**Fig. 1:**
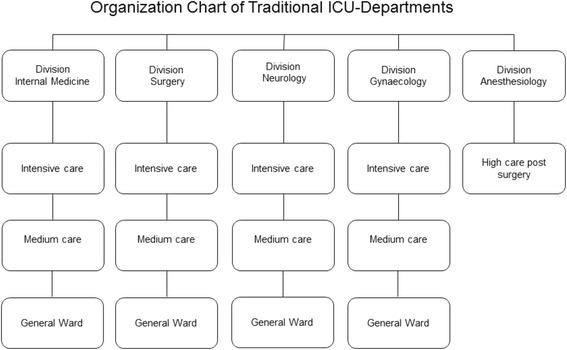
The traditional ICU model. Organizational chart of the intensive care departments designed vertical based on the medical specialty

**Fig. 2:**
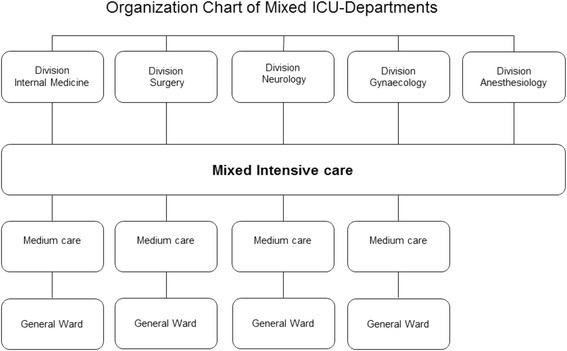
The mixed ICU model. Organization chart of the intensive care departments designed horizontally based on the intensity of care

In manufacturing and business, one source of competitive advantage is consolidation of separate units which results in increase of scale and reduction of overhead. Within the service industry, consolidation or pooling also results in improvement of service capacity, hence reduction of wait time. The positive effect of pooling or consolidation might be a future improvement within the ICU. The formation of a mixed closed format ICU model: one central ICU in a hospital instead of several small ones (traditional closed format ICU model), to which all patients independent of their underlying conditions are admitted. This is illustrated in Figs. 1 and 2. We hypothesize that, overall, a mixed closed format ICU model improves patient outcome, increased admission capacity, and reduces at the same time costs, compared to a traditional closed format ICU model; however, data to support this hypothesis is lacking. Studies should aim to obtain data on both patient outcome and cost, before and after introduction of a mixed closed format ICU model.

#### Step down unit

The main purpose of a step down unit on an ICU is to bridge the differences between the ICU and a general ward (but not to function as a medium care, where “step-up” is also allowed) and therefore to reduce the readmission ratio. Readmission is known to be associated with adverse outcomes [34]. Caregivers and patients experience a substantial gap in monitoring and level of care which may lead to a reduction of the threshold for readmission. Respiratory insufficiency remains one of the most important reasons for readmission. A prospective study is needed to determine whether a step down unit is able to decrease readmission rates.

#### The mobile intensive care unit

Inter-hospital transport of critically ill patients may have a positive effect on resource utilization by pooling admission capacity of the medical centers or creating focus clinics for certain patient categories. Furthermore, transportation enables the distribution of the most severe patients to large referral medical centers and the less severe patients to more rural centers. In this way, you optimize the utilization by level of care and it may improve the overall patient outcome [35]. For instance, the transportation of patients with very severe respiratory failure, which was considered to preclude conventional ventilation for safe transfer to tertiary centers, was managed by an extra corporal membrane oxygenation referral and retrieval program in New South Wales and had a high rate of survival [36]. The possible downside of these inter-hospital transports is the exposure to adverse events and the cost. However, when using a mobile intensive care unit (MICU) compared to a normal ambulance, a reduction of adverse events during inter-hospital transport from 34 to 12.5% was achieved [37]. Furthermore, it is suggested that the condition of the patient does not predict the risk of adverse events but the formation of the team and equipment available [38]. Unfortunately, data on patient outcome and cost are lacking to support the possible upside on resource utilization improvement as mentioned above.

#### Distant monitoring—telemedicine

The closed format model of ICU is a superior clinical practice as discussed above, but shortage of intensivists and financial reasons led to the development of telemedicine. In one study [39], they showed that the implementation of telemedicine resulted in a relative risk reduction of mortality of 0.73 (95%CI 0.55–0.95) and reduction of ICU stay from 4.35 to 3.63 days. Furthermore, this resulted in lower variable cost per patient and higher hospital revenues from increased case volumes. Although not all other studies [40–43] have shown similar effects, in general, the results suggest that telemedicine might be a solution to bridge the shortage of intensivists while still improving quality of care and reducing health-care cost in rural areas [44]. Of note, there is no data providing evidence that implementing telemedicine in rural areas is superior to transporting the critically ill patients to appropriate staffed ICUs. Furthermore, no studies are available to support telemedicine for ICUs located in high-density populations.

#### Total quality management and quality indicators

Total quality management (TQM) is a philosophy of management for continuously improving the quality of products and processes. According to TQM, quality is a process that can be managed and requires ongoing evaluation and change (continuous quality improvement (CQI)). Before we can apply TQM and CQI in the ICU, we need specific quality indicators. A few of these indicators have been identified for the ICU: (1) percentage of patients with central venous catheter infections, (2) percentage of patients with ventilator-associated pneumonia, (3) percentage of patients with vancomycin-resistant enterococcus, (4) number of complications per patient, (5) rate of gastrointestinal bleeding, (6) average days of mechanical ventilation, (7) average ICU length of stay, and (8) patient satisfaction [45]. Other interesting performance measures for both the health-care provider and the patient are hospital mortality and ICU mortality, corrected for expected mortality (standardized mortality ratio (SMR)) and readmission rates. Obviously, some indicators may influence other ones, e.g., LOS and readmission rate or duration of mechanical ventilation and number of reintubations. Recently, it is recognized that quality indicators (QIs) should be actionable. They should be developed in such a way that they can be influenced by, in the case of use in intensive care medicine, intensivists. By developing new QIs that are actionable, reliable, valid, and easy to register, quality improvement in intensive cares will be supported. Using a modified RAND technique, new QIs for blood use at Dutch ICUs were developed [45]. The set of QIs will be part of the National Intensive Care Evaluation [46]. To apply TQM and QI, it cannot be neglected to mention evidence-based practice. Indeed, nowadays, quality improvements need to be shown to be effective in clinical studies before they become widely adopted. Although TQM and CQI work in the business environment, it may not be optimal for the health-care industry. A critique on both TQM and CQI is the narrow focus on outcome and not the underlying process.

#### Use of safety management systems in the ICU and risk assessment

From recent studies in ICU patients, we know that improving patient safety has impact on the safety climate as well as on mortality and length of stay [47, 48]. Risk management is an important part of the safety management system. Retrospective risk analysis focuses on incidents that have taken place; prospective risk analysis has the advantage of its true preventive nature. Retrospective methods that can be used are, for instance, root cause analysis and tripod beta analysis. For prospective risk analysis, there are several other methods. Examples are the bow-tie method and the health-care failure mode and effect analysis (HFMEA) [49–51].

#### Lean management/Toyota Production System

The processes in an ICU can partly be seen as a manufacturing process. A future improvement could be identifying processes which work in the manufacturing industry which are also applicable for the ICU. One of the possible processes could be lean management. Lean management is a philosophy which is mainly derived from the Toyota Production System (TPS). The original seven “wastes” also known as “muda” were identified by Taiichi Ohno, the Toyota executive felt to be the main developer of the Toyota Production System [52]. Later, on the eighth waste, “talent” has been added. These eight manufacturing terms are presented in Table 2 and can easily be translated into health-care organizations which were done by John Toussaint and Roger Gerard, in their book, *On the Mend* [53]. They have identified “the eight wastes of lean healthcare.” Lean is not a “fix it and forget it” approach. It takes a constant commitment from leadership to allow the culture and the processes to create better approaches and more efficient pathways. Lean is not easy to implement and sustain as there is not one common recipe. Lean has succeeded in a number of health-care circumstances [54] but failed in many [55]. Evidence for lean management in the ICU is scarce; only a few projects have been published specifically targeting the ICU department [56, 57]. Whether lean will become important in ICU organizations will depend mainly on the willingness to let lean enter the ICU and the ability to implement and keep improving lean processes.

**Table 2:** Comparison of application of lean management in manufacturing and health-care organization

Type of problem	Manufacturing organization	Health-care organization	Implication for intensive care
Overproduction	Producing ahead of need	Unnecessary treatment and overuse of diagnostic testing	Clear treatment goals and end-of-life decision guidelines
Waiting	Operators standing idle waiting for other workers or machines to finish	Patient waits for an appointment, for test results, for a bed, for discharge paperwork	Clear admission and discharge guidelines
Transport	Moving parts and products unnecessarily	Taking patients to and from tests, moving patients from one room to another	Diagnostic tests being performed at bed side
Over Processing	Performing unnecessary or incorrect activities	Unnecessary forms, asking the same patient the same question more than once, charting everything instead of charting by exception	Digital system Preventing re-enter of patient data Patient centric rounding
Inventory	Having more than the minimum stock necessary	Overstocked drugs that expire, under stocked surgical supplies that lead to delays while staff search for them	Pooling of inventories within the hospital or even within the region just in time
Motion	Making workers look for parts, tools, documents, etc	Searching for supplies, forms, drugs	Correct and logic labelling of all supplies, forms, and drugs
Defects	Inspection, rework, and scrapping parts that do not meet standards	Making and correcting errors, checking for errors	Clear protocols including feedback mechanisms and e-alerts
Talent Waste	Failure to listen to employee ideas for improvement	Using highly trained individuals to do jobs that could be performed by less expensive personnel, failure to listen to employee ideas for improvement	Focus on ICU physician and ICU nurse specific tasks and outsource tasks such as washing patients, paperwork, and move tasks down from ICU physician to ICU nurse when possible

#### Compliance

The evidence that changes in organizational structures in the intensive care department are able to have significant impact on patient outcome and cost is straightforward and sound. However, the implementation and compliance with these recommendations can be difficult. Pronovost et al. showed [58] that the closed format was not implemented in all hospitals and, when so, the intensivists were often not empowered to make decisions. Hence, on paper, the hospitals fulfil the criteria but, in practice, not much has changed. This study underlines the importance of clearly defining guidelines and the importance of implementing processes. Although evidence exists, hospitals and physicians may be reluctant to implement these processes. Explanations for this may be reluctance to change, disbelief of the results of studies, and local factors making it impossible to extrapolate the results in the hospital. In the future, resources should not only be devoted to identifying new process improvements but also to implementing proven processes. Whether this is a role for the physicians, the government, or external companies remains to be determined.

## Conclusions

The organization of the intensive care department has been changed over the past decades resulting in better patient outcome and reduction of cost. Major changes are the implementation of the closed format and electronic patient record. Challenges are ahead as the ICU is taking up the largest share of national health-care expenditure, and with the aging of the population, this will continue to increase. We would like to advocate for standard inclusion of cost analysis into future study reports, as financial constraints within the health-care industry have become an important issue nowadays and cost-effectiveness may influence decision-making whether or not to implement an intervention. Besides future improvements of organizational structures within the ICU, the focus should also be on implementation of and compliance with proven beneficial organizational structures.

## Abbreviations

HFMEA: Health-care failure mode and effect analysis
ICU: Intensive care unit
LOS: Length of stay
MERIT: Medical early response intervention and treatment
MICU: Mobile intensive care unit
PDMS: Patient digital management systems
QI: Quality indicators
SMR: Standardized mortality ratio
SWIFT: Stability and workload index for transfer
TPS: Toyota Production System
TQM: Total quality management
